# Impact of early tacrolimus exposure on outcomes after allogeneic hematopoietic cell transplantation for acute myeloid leukemia and myelodysplastic syndromes

**DOI:** 10.1007/s00277-026-06988-0

**Published:** 2026-04-09

**Authors:** Paul Jäger, Andreas Alexander Ralfs, Felicitas Schulz, Nora Liebers, Jonathan Bobak, Ben-Niklas Baermann, Sören Twarock, Alexander Hölscher, Kathrin Nachtkamp, Annika Kasprzak, Joonatan Lavikka, Felix Matkey, Titus Watrin, Malika El Yaouti, Ulrich Germing, Sascha Dietrich, Guido Kobbe

**Affiliations:** 1 Department of Hematology, Oncology and Clinical Immunology, Medical Faculty and University Hospital Düsseldorf, Heinrich Heine University Düsseldorf, Düsseldorf, Germany; 2 Institute of Translational Pharmacology, Medical Faculty and University Hospital Düsseldorf, Heinrich Heine University Düsseldorf, Düsseldorf, Germany; 3 Department of Hematology, Oncology and Clinical Immunology, University of Duesseldorf, Medical Faculty, Moorenstr. 5, Duesseldorf, 40225 Germany

**Keywords:** Tacrolimus, Allogeneic hematopoietic cell transplantation, Graft-versus-host disease, Acute myeloid leukemia, Myelodysplastic syndromes, Pharmacokinetics, Immunosuppression

## Abstract

Background. Calcineurin inhibitors balance graft-versus-host disease (GvHD) prevention against graft-versus-leukemia (GvL) effects after allogeneic hematopoietic cell transplantation (allo-SCT). Whether the magnitude of early tacrolimus exposure influences relapse or survival remains unclear. Methods. We retrospectively studied 122 adults with AML (n = 80) or MDS (n = 42) undergoing 10/10 matched-unrelated donor (MUD) allo-SCT from 2014 to 2021. All received rabbit anti-thymocyte globulin (ATG), tacrolimus and mycophenolate mofetil (MMF) for GvHD Prophylaxis. Tacrolimus exposure was quantified as area under the concentration–time curve over days 1–30 (AUC30) and 1–100 (AUC100). Primary endpoints were overall survival (OS) and event-free survival (EFS); secondary endpoints were cumulative incidence of relapse (CIR), non-relapse mortality (NRM), and GvHD. Analyses included median splits, quartiles/deciles, competing-risks models, Cox regression, and smoothed 5-year KM estimates across continuous AUC30. Patients with relapse/death before day + 30/+100 were excluded from the respective AUC analyses. Results. Median follow-up was 61 months; 5-year OS and EFS were 68% and 43%. Neither AUC30 nor AUC100 (median split or quartiles) was associated with OS, EFS, CIR, or NRM. In multivariable models, tacrolimus exposure was not significant; worse outcomes correlated with older age and female donor for OS, and with AML diagnosis and no CR at allo-SCT for EFS. Smoothed survival across continuous AUC30 showed no meaningful gradient. Any-grade and severe aGvHD, and overall/grade ≥ III chronic GvHD, were comparable across exposure groups. Conclusions. Within a homogeneous MUD/ATG/MMF setting, early tacrolimus exposure did not independently predict relapse, NRM, EFS, or OS. Patient and donor factors outweighed tacrolimus AUC. Findings argue against AUC-targeting to improve outcomes; risk-adapted tapering and MRD-guided interventions may offer greater leverage.

**Graphical abstract:**

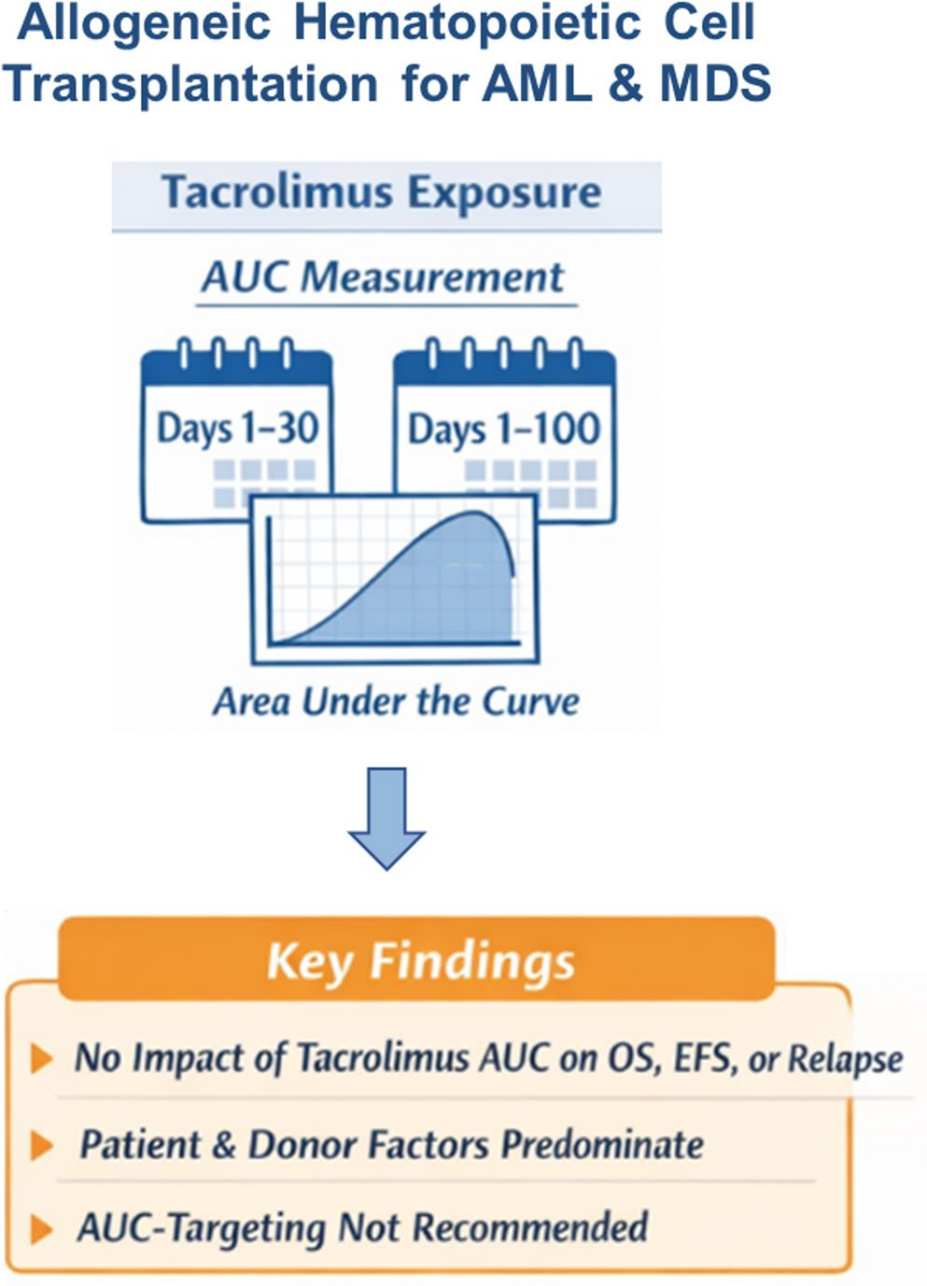

**Supplementary Information:**

The online version contains supplementary material available at 10.1007/s00277-026-06988-0.

## Introduction

Allogeneic stem cell transplantation (allo-SCT) remains the only curative option for many patients with acute myeloid leukemia (AML) and myelodysplastic syndromes (MDS) [1]. Its efficacy relies on the graft-versus-leukemia (GvL) effect, whereby donor-derived immune cells eliminate residual malignant clones [2]. However, allo-SCT is complicated by graft-versus-host disease (GvHD), a leading cause of morbidity and mortality, particularly within the first 100 days [3].

To mitigate GvHD while preserving GvL, calcineurin inhibitors (CNIs) such as cyclosporine A (CSA) or tacrolimus are standard, often combined with mycophenolate mofetil (MMF) or methotrexate [4]. Higher CNI exposure reduces GvHD but may attenuate GvL and increase relapse risk [5]. Elevated CSA levels have been associated with increased relapse after allo-SCT [6–8], underscoring the balance between immunosuppression, relapse prevention, and non-relapse mortality (NRM).

Both cyclosporine A (CSA) and tacrolimus inhibit calcineurin signaling and thereby suppress T-cell activation through inhibition of NFAT-dependent IL-2 transcription. However, tacrolimus is generally considered more potent and shows distinct pharmacokinetic characteristics compared with CSA, including different exposure variability patterns under therapeutic drug monitoring [5]. Consequently, observations from CSA-based studies regarding exposure-dependent relapse risk may not directly translate to tacrolimus, highlighting the need to specifically evaluate tacrolimus exposure in contemporary transplant platforms.

At our center, CSA is mainly used for HLA-identical sibling donor allo-SCT, whereas tacrolimus is preferred for 10/10 matched-unrelated donor (MUD), reflecting a higher baseline GvHD risk. Despite the widespread use of tacrolimus, systematic data on how cumulative exposure influences relapse, survival, and NRM remain limited. Clinically, lower tacrolimus trough levels are sometimes used in patients deemed at high relapse risk to preserve GvL, but this strategy lacks robust prospective validation.

Residual disease burden and genomic risk strongly predict post-transplant outcomes [9]. Craddock et al. (2010) further suggested that immunosuppressive intensity may interact with measurable residual disease (MRD) to shape the risk of relapse and the chance of survival. These observations raise the question of whether tacrolimus exposure differentially affects relapse control across patient subgroups.

We therefore retrospectively analyzed AML and MDS patients undergoing allo-SCT from HLA-matched unrelated donors at our institution. Tacrolimus exposure during the first 100 days post-transplant was quantified and correlated with overall survival (OS), event-free survival (EFS), relapse, and GvHD. Our aim was to determine whether the magnitude of tacrolimus exposure influences the balance between GvL, GvHD, and NRM, and whether specific subgroups might benefit from tailored immunosuppression.

## Methods

### Study population and transplant procedures

**Fig. 1:**
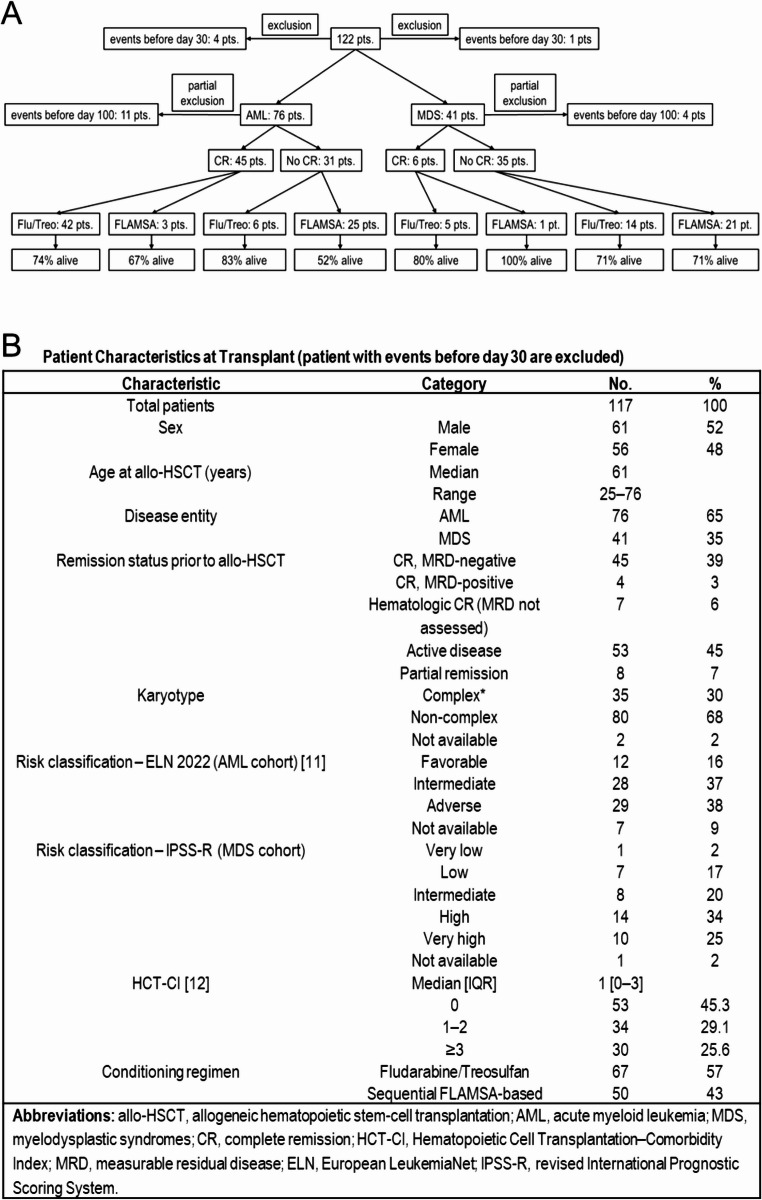
**A** CONSORT diagram. AML/MDS patients undergoing 10/10 HLA-matched unrelated donor allo-SCT (2014–2021). Patients who relapsed or died before day 30 (for the 30-day analysis) or before day 100 (for the 100-day analysis) were excluded to enable consistent assessment of tacrolimus exposure over the respective interval. **B** Patient characteristics at transplant [11, 12]

We retrospectively analyzed 122 adults with AML (*n* = 80) or MDS (*n* = 42) who underwent allo-SCT from 10/10 MUD at the University Hospital Düsseldorf between 2014 and 2021. The median age at transplant was 61 years. Conditioning regimens consisted of either sequential FLAMSA-based approaches (fludarabine, cytarabine, amsacrine followed by a conditioning backbone) or non-sequential regimens. Sequential FLAMSA-based regimens were typically combined with reduced-intensity conditioning backbones such as melphalan (100–150 mg/m²) or treosulfan (10 g/m²) and were mainly applied in patients with active or high-risk disease. Non-sequential conditioning predominantly comprised fludarabine/treosulfan (most commonly treosulfan 14 g/m²), representing a reduced-toxicity platform that may approach myeloablative intensity depending on dosing. Given this overlap, conditioning was categorized by regimen type rather than by strict myeloablative versus reduced-intensity classification (Fig. 1A). All patients received rabbit anti-thymocyte globulin (ATG) (cumulative 30 mg/kg), tacrolimus, and MMF until day + 42. 

### Exposure definition and exclusions

Tacrolimus was initiated on day − 1 according to institutional practice at a starting dose of approximately 0.05 mg/kg twice daily, with therapeutic drug monitoring performed in parallel. From around day + 1 onward, the dose was typically reduced to approximately 0.03 mg/kg twice daily and subsequently adjusted based on trough concentrations, renal function, and clinical tolerability. During the early post-transplant period, target trough concentrations were generally maintained at approximately 10–12 ng/mL during the first 2–3 weeks, followed by lower target levels of approximately 5–8 ng/mL thereafter.

Tacrolimus dosing was guided by routine trough level monitoring as part of standard clinical care in the allo-SCT setting, whereas formal AUC-guided dosing was not performed. For this retrospective analysis, tacrolimus exposure was quantified as the area under the concentration–time curve (AUC; ng·day/mL) over days 1–30 (AUC30) and days 1–100 (AUC100). Concentration–time curves were reconstructed from serial trough measurements using linear interpolation between consecutive values, and AUCs were calculated using the trapezoidal rule (GraphPad Prism). This approach provides a pragmatic estimate of cumulative tacrolimus exposure under routine inpatient and outpatient monitoring rather than full pharmacokinetic profiling.

Patients who relapsed or died before day + 30 (for AUC30 analyses) or before day + 100 (for AUC100 analyses) were excluded to ensure consistent exposure assessment over the respective intervals (Fig. 1B).

### Endpoints

Primary endpoints were overall survival (OS; time from transplant to death from any cause) and event-free survival (EFS; time to relapse or death). Secondary endpoints included the cumulative incidence of relapse (CIR), NRM, and GvHD. Acute GvHD was graded according to the modified Glucksberg criteria, and chronic GvHD according to NIH consensus criteria. Due to the retrospective nature of the study, lower-grade manifestations were not consistently documented in sufficient detail for reliable severity stratification. Therefore, GvHD was primarily analyzed as overall occurrence, with additional analyses focused on clinically robust endpoints, namely grade ≥ III acute GvHD and severe chronic GvHD. 

### Statistical analysis

We summarized continuous variables by median and interquartile range (IQR) and categorical variables by counts and percentages. Survival was estimated by Kaplan–Meier and compared using the log-rank test. For competing-risks endpoints (CIR and NRM), cumulative incidence functions were compared using the Gray test; where indicated, sensitivity analyses aligned NRM flags with death status. Multivariable associations with OS and EFS were assessed using Cox proportional hazards models (IBM SPSS Statistics; Cox regression with Efron ties), with covariates selected a priori based on clinical relevance and prior evidence. To account for the limited number of events, the number of variables included in the models was restricted to avoid overfitting. Results are reported as hazard ratios (HR) with 95% confidence intervals (CI) and Wald p-values; global model χ² statistics are provided. To visualize the functional form of the association between continuous AUC30 and survival, we generated non-parametric smoothed 5-year Kaplan–Meier estimates across the AUC30 distribution using rolling nearest-neighbor windows (~ 20–25% of the cohort), plotting estimated 5-year OS/EFS as a function of AUC30. Grouped comparisons used median splits and, where stated, quartiles/deciles of AUC.

All analyses used two-sided tests with *p* ≤ 0.05 considered statistically significant (0.05–0.10 interpreted as a trend). The study was approved by the ethics committee of the University Hospital Düsseldorf (reference 2023–2538). The study was conducted in accordance with the Declaration of Helsinki.

## Results

### Cohort and follow-up; primary 30-day exposure analysis

**Fig. 2:**
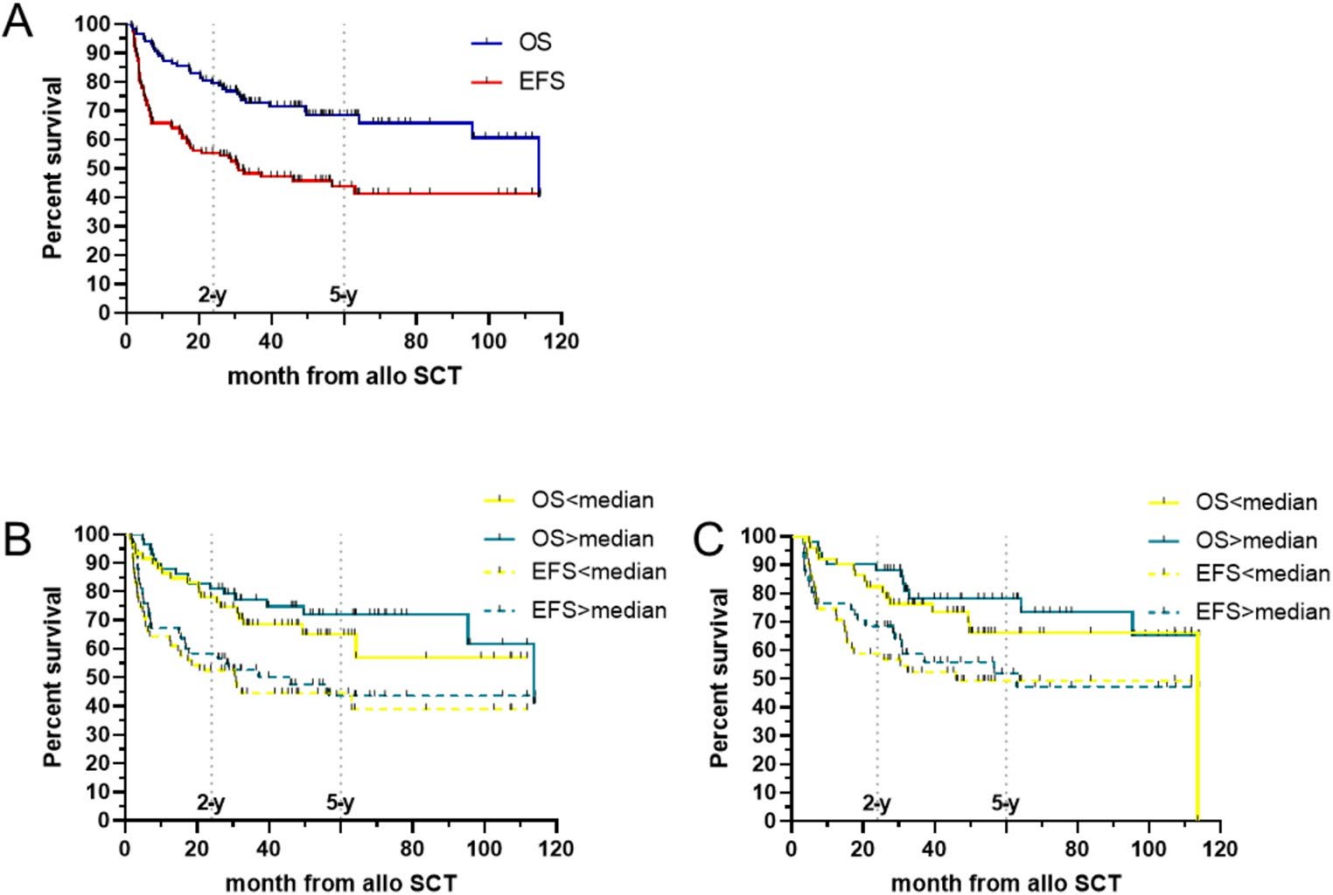
Survival after allo-SCT in AML/MDS.** A** Entire cohort (*n* = 122): Kaplan–Meier curves for overall survival (OS, solid blue) and event-free survival (EFS, solid red). Dotted vertical lines mark 2- and 5-year time points. **B** Survival by 30-day tacrolimus exposure and **C** survival by 100-day exposure, each dichotomized at the cohort median: OS (solid) and EFS (dashed) for AUC < median vs. AUC ≥ median

The median follow-up, calculated using the reverse Kaplan–Meier method, was 61 months (range, 1.4–114.1 months). The 5-year (5-y) OS and EFS rates of the entire cohort were 68% and 43%, respectively (Fig. 2A). Tacrolimus was administered for a median of 172 days (range, 44–1089 days). The shortest treatment durations corresponded to patients with early events before day 100 and were therefore excluded from the 100-day analyses, whereas the longest durations were observed in patients who subsequently developed chronic GvHD. To ensure sufficiently large groups for comparison, we initially focused on the 30-day tacrolimus AUC, dichotomizing at the median (322.5 ng·d/mL). No significant differences in OS or EFS were observed, with a 5-y OS of 72% for AUC ≥ median and 65% for AUC < median; the 5-y EFS was 44% in both groups (Fig. 2B). Stratification into quartiles and comparison of the lowest and highest quartiles versus the remainder likewise showed no statistically significant differences in OS or EFS (data not shown).

### Sensitivity analyses: 100-day exposure; exploratory extremes

**Fig. 3:**
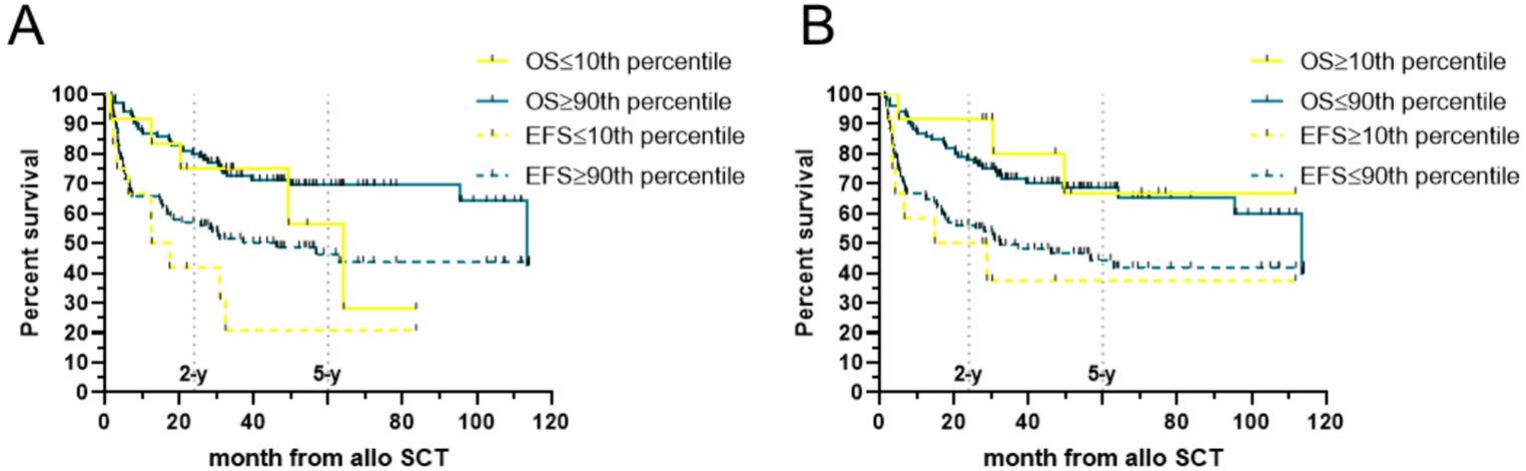
Survival by extreme early tacrolimus exposure.** A** Based on 30-day exposure (AUC30): overall survival (OS) (solid) and event-free survival (EFS) (dashed) comparing the lowest decile (≤ 282.7 ng·d/mL; yellow) vs. the remainder (teal). **B** OS and EFS comparing the highest decile (≥ 374.6 ng·d/mL; yellow) vs. the remainder (teal)

We next evaluated whether tacrolimus exposure during the first 100 days influenced survival or relapse risk. For these analyses, patients with a documented relapse within the first 100 days were excluded to avoid confounding by indication (prompt IS reduction). The 100-day AUC showed no significant associations with outcome: 5-y OS was 66% for AUC < median versus 78% for AUC ≥ median, and 5-y EFS was 49% versus 52%, respectively (Fig. 2C). Quartile analyses again revealed no significant differences in OS, EFS, CIR, or NRM (data not shown). In an exploratory analysis at day + 100, patients in the lowest AUC100 decile (≤ 10th percentile; AUC100 ≤ 750.8 ng·d/mL; *n* = 11) showed numerically inferior outcomes compared with the remainder of the cohort (OS log-rank *p* = 0.039; EFS *p* = 0.008; data not shown), whereas very high exposure (≥ 90th percentile) was not associated with differences in outcome. These findings should be interpreted with caution given the small sample size and potential confounding by indication. Because extremely low exposure clustered in high-risk subsets (e.g., AML, induction failure, adverse cytogenetics)—potentially prompting lower tacrolimus levels—we also examined 30-day AUC, where post-transplant, risk-driven dose adjustments are less likely to bias exposure. Patients in the lowest decile (AUC30 ≤ 282.7 ng·d/mL; *n* = 12) showed numerically lower 5-y OS and EFS than the highest decile, but differences were not statistically significant (OS *p* = 0.36; EFS *p* = 0.13; Fig. 3A–B). Consistent with prior work on early CSA exposure, we therefore used the 30-day AUC as the primary exposure for subgroup and multivariable analyses.

### Multivariable models and smoothed survival

In multivariable Cox models, AUC30 ≥ median was not associated with OS (HR 0.80; *p* = 0.523) or EFS (HR 0.91; *p* = 0.723). Age ≥ median (HR 2.76; *p* = 0.005) and female donor (HR 2.95; *p* = 0.002) were independently associated with worse OS, while AML versus MDS did not reach statistical significance (HR 1.55; *p* = 0.246). For EFS, AML versus MDS (HR 1.99; *p* = 0.040) and absence of complete remission before transplant (HR 1.80; *p* = 0.038) were adverse factors; high-risk compound score, HCT-CI ≥ 3, and age ≥ median were not significant, and female donor showed a borderline association (HR 1.66; *p* = 0.077).

In addition to grouped analyses, tacrolimus exposure was examined as a continuous variable. In Cox proportional hazards models with AUC30 entered continuously (per 50 ng·day/mL increase), AUC30 was not associated with OS or EFS (both HRs close to 1.0; *p* > 0.2). Modelling AUC30 using restricted cubic splines showed no evidence for a non-linear association with OS or EFS (p for non-linearity > 0.2).

**Fig. 4:**
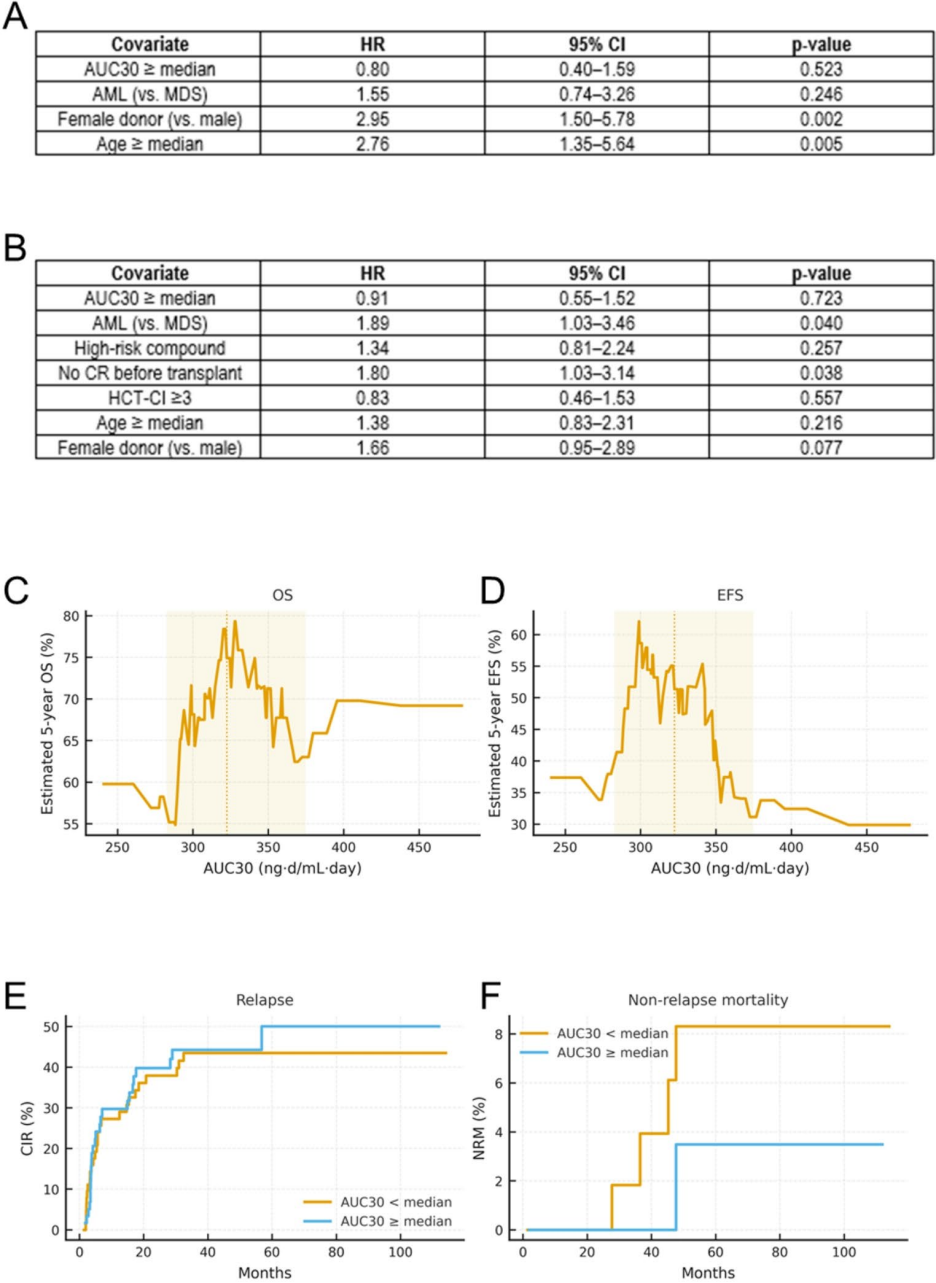
Multivariable models, smoothed survival by AUC30, and competing risks. **A** Overall survival (OS): Cox proportional hazards model (*n* = 117; events = 37). Hazard ratios (HR) with 95% confidence intervals (CI) and Wald p-values are shown. Global model: χ²=18.43 (df = 4), *p* = 0.001. **B** Event-free survival (EFS): Cox model (*n* = 117; events = 63). HRs with 95% CI and Wald p-values are shown. Global model: χ²=12.81 (df = 7), *p* = 0.077. Note: HR > 1 indicates increased hazard. **C–D** Smoothed 5-year KM estimates across continuous AUC30: **C** OS and **D** EFS plotted against 30-day tacrolimus AUC (AUC30) using rolling nearest-neighbor windows (~ 25% of the cohort). The dotted vertical line marks the cohort median (322.5 ng·d/mL), and the shaded band indicates the 10th–90th percentile range. **E–F** Competing-risk analyses by 30-day AUC median split: **E** cumulative incidence of relapse (CIR) and **F** non-relapse mortality (NRM), estimated with competing-risks methods (median AUC30 as cutoff)

Smoothed 5-year survival estimates across the continuous AUC30 range showed no meaningful gradient for OS or EFS (Fig. 4C–D), consistent with the absence of an AUC effect in the Cox models. In competing-risks analyses using the 30-day AUC median split, 5-year CIR was similar across groups and NRM was low in sensitivity analyses aligning NRM with death status (Fig. 4E–F); after adjustment, tacrolimus exposure was not a significant predictor of CIR or NRM.

### GvHD outcomes and safety/immune reconstitution

Overall, acute GvHD occurred in 45 of 122 patients (36.9%), including 34 patients with classical acute GvHD and 11 with late-onset acute GvHD. Severe acute GvHD (grade ≥ III) was observed in 4 patients (3.3%). Chronic GvHD developed in 17 evaluable patients (13.9%), with severe chronic GvHD observed in 4 patients.

Early tacrolimus exposure showed no discernible association with GvHD incidence or severity. With the 30-day AUC median split, cumulative-incidence curves for any-grade acute GvHD overlapped, and severe acute GvHD was rare in both groups (Gray test n.s.; data not shown). Quartile analyses yielded a similar pattern without evidence of a dose–response relationship. Results were comparable for chronic GvHD, with no consistent differences across exposure groups.

Finally, across the 30-day AUC median split, there were no clinically relevant differences in bilirubin, creatinine (early or peak), fragmentocytes, tacrolimus duration, or time to CD4 reconstitution; only the expected higher mean tacrolimus level over days 1–100 was observed in the high-AUC group (Supplementary Table 1). 

## Discussion

Across multiple specifications—median split, quartiles/deciles, continuous (splines), and competing-risk regression— the magnitude of tacrolimus exposure during the first 30 or 100 days did not independently affect relapse, NRM, EFS, or OS in uniformly treated patients at our center. Patient and donor factors (age, female-to-male donor sex), disease risk, and pre-transplant remission status were stronger and consistent determinants of outcome, overshadowing any putative AUC effect [1, 9]. In contrast to cyclosporine, where higher exposure has been linked to more relapse [5–8, 10], tacrolimus may exert more potent and steadier calcineurin inhibition at standard practice levels, leaving relatively little actionable “dynamic range” in troughs/AUC within routine care [3, 4]. This pharmacologic ceiling—together with therapeutic drug monitoring that narrows variability—could explain the absence of a reproducible exposure–response signal in our cohort.

Confounding by indication remains possible despite our design choices (exclusion of early relapse for AUC100, primary focus on AUC30, multivariable and competing-risk models), as very low tacrolimus exposure clustered in biologically adverse subsets (e.g., AML with induction failure or poor-risk cytogenetics), where clinicians may have intentionally favored lower immunosuppression to preserve graft-versus-leukemia effects [7]. The observation of inferior outcomes in the lowest AUC100 decile should therefore be interpreted with caution, as these patients disproportionately represented high-risk disease subsets and the small number of patients in these tail groups limits the robustness of these findings. Accordingly, analyses of exposure extremes should be considered exploratory and hypothesis-generating rather than indicative of a causal relationship. Similarly, sequential FLAMSA-based regimens were preferentially used in patients with higher-risk disease, limiting the interpretability of conditioning-related effects.

The relatively low incidence of chronic GvHD observed in our cohort may reflect the consistent use of ATG-based GvHD prophylaxis, which is known to reduce cGvHD rates, as well as the retrospective nature of data collection. In particular, lower-grade manifestations may be underreported in retrospective datasets. Accordingly, our analyses focused on overall GvHD occurrence and clinically robust endpoints.

Our findings suggest that, within a standardized MUD/ATG/MMF transplant platform, early tacrolimus exposure may have limited influence on long-term outcomes. However, given the retrospective design, potential selection biases, and the limited number of patients at the extremes of exposure, these results should be interpreted with caution.

In high-risk disease, we would prioritize risk-adapted immunomodulation rather than aiming for tacrolimus AUC thresholds: timely tapering of tacrolimus in the absence of GvHD, MRD-guided pre-emptive donor lymphocyte infusions (DLI), and maintenance strategies (e.g., hypomethylating agents; targeted inhibitors in molecularly defined AML) to augment GvL without increasing calcineurin inhibitor intensity [2, 7]. Immunologic stimulation, including structured vaccination schedules post-SCT, may contribute to immune reconstitution and disease control but should be used within guideline frameworks and clinical trials [3].

Strengths of our study include a homogeneous donor setting (10/10 MUD), standardized GvHD prophylaxis, prespecified analyses (median, quartiles/deciles, continuous splines), and concordant results across OS/EFS and CIR/NRM. Limitations are the retrospective single-center design, potential measurement imprecision in AUC reconstruction, limited MRD availability, and small numbers at exposure extremes—precluding firm causal inference.

Prospective studies should test AUC-guided vs. standard tacrolimus with MRD-stratification, incorporate time-in-therapeutic-range/variability metrics, and explore sex-mismatch–adapted prophylaxis schemas [3, 9]. Until such data exist, optimizing patient/donor selection, early MRD-informed taper/DLI, and maintenance therapy likely offers greater leverage than fine-tuning tacrolimus exposure.

## Supplementary material

Below is the link to the supplementary material.

Supplementary Table 1 Toxicity, laboratory parameters, tacrolimus exposure, and immune reconstitution by 30-day tacrolimus AUC (median split). Values are median [IQR]; p values from two-sided Mann–Whitney U tests. AUC30 median = 322.5 ng·d/mL·. As expected, the high-AUC group showed a higher 100-day mean tacrolimus level; all other parameters showed no material between-group differences.

## Data Availability

The data supporting the findings of this study are available from the corresponding author upon reasonable request.
