# Supplementary material for: Impact of early tacrolimus exposure on outcomes after allogeneic hematopoietic cell transplantation for acute myeloid leukemia and myelodysplastic syndromes
Source: Ann Hematol. 2026 Apr 9;105(5):232. doi: 10.1007/s00277-026-06988-0 (PMC13065609; doi:10.1007/s00277-026-06988-0)
Supplement: Supplementary file 1 — Toxicity, laboratory parameters, tacrolimus exposure, and immune reconstitution by 30-day tacrolimus AUC (median split). Values are median [IQR]; p values from two-sided Mann–Whitney U tests. AUC30 median = 322.5 ng·d/mL·. As expected, the high-AUC group showed a higher 100-day mean tacrolimus level; all other parameters showed no material between-group differences. [file 277_2026_6988_MOESM1_ESM.docx]

**
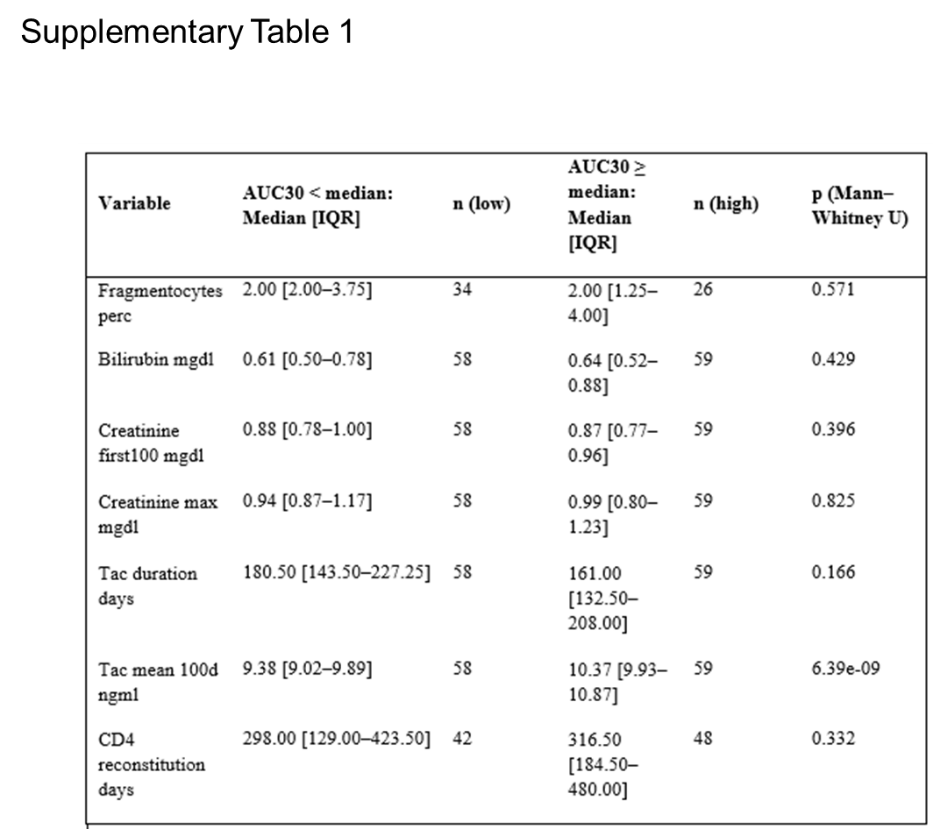
**

**Supplementary Table 1.** Toxicity, laboratory parameters, tacrolimus exposure, and immune reconstitution by 30-day tacrolimus AUC (median split). Values are median [IQR]; p values from two-sided Mann–Whitney U tests. AUC30 median = 322.5 ng·d/mL·. As expected, the high-AUC group showed a higher 100-day mean tacrolimus level; all other parameters showed no material between-group differences.
